# Multi-stage sleep classification using photoplethysmographic sensor

**DOI:** 10.1098/rsos.221517

**Published:** 2023-04-12

**Authors:** Mohammod Abdul Motin, Chandan Karmakar, Marimuthu Palaniswami, Thomas Penzel, Dinesh Kumar

**Affiliations:** ^1^ Department of Electrical and Electronic Engineering, Rajshahi University of Engineering and Technology, Kazla, Rajshahi 6204, Bangladesh; ^2^ School of IT, Deakin University, Burwood, Melbourne, VIC 3125, Australia; ^3^ Department of Electrical and Electronic Engineering, The University of Melbourne, Melbourne, VIC 3010, Australia; ^4^ Interdisciplinary Sleep Medicine Center, Charite Universitatsmedizin, 10117 Berlin, Germany; ^5^ School of Electrical and Biomedical Engineering, RMIT University, Melbourne, VIC 3001, Australia

**Keywords:** multi-stage sleep classification, wearable PPG signal, cardio-respiratory biomarker, arterial blood pressure, pulse rate variability, support vector machine

## Abstract

The conventional approach to monitoring sleep stages requires placing multiple sensors on patients, which is inconvenient for long-term monitoring and requires expert support. We propose a single-sensor photoplethysmographic (PPG)-based automated multi-stage sleep classification. This experimental study recorded the PPG during the entire night's sleep of 10 patients. Data analysis was performed to obtain 79 features from the recordings, which were then classified according to sleep stages. The classification results using support vector machine (SVM) with the polynomial kernel yielded an overall accuracy of 84.66%, 79.62% and 72.23% for two-, three- and four-stage sleep classification. These results show that it is possible to conduct sleep stage monitoring using only PPG. These findings open the opportunities for PPG-based wearable solutions for home-based automated sleep monitoring.

## 1. Introduction

Healthy sleep is necessary as it supports brain and body restoration [1]. Nevertheless, nearly one-third of the population suffers from chronic sleep disturbances resulting in a number of physiological and psychological problems [2]. Sleep has five stages; of these, four have non-rapid eye movement (NREM), and one is rapid eye movement (REM). Healthy sleep goes through these stages cyclically, but disturbed sleep alters this cycle. Sleep stages are influenced by autonomic nervous system functions [3]. Sleep stages are identified to investigate the quality of sleep using a multi-sensors system called polysomnography (PSG). This requires the recording of various physiological signals such as electroencephalogram (EEG), electrocardiogram (ECG), electrooculogram (EOG), electromyogram (EMG), airflow, arterial oxygen saturation and respiratory effort [4] during sleep. However, PSG-based sleep analysis is limited to the purpose-built sleep laboratories and requires a significant time of the experts, leading to extensive waiting time for patients. Another major shortcoming is that these tests are intrusive and therefore, may disturb the normal sleep of the patient.

Home-based computerized sleep analysis will facilitate several people. However, this is highly challenging because many sensors need to be carefully placed, and incorrect placement can result in many artefacts in the recordings [4]. Multi-modal approaches with multiple physiological sensors, in general, have higher accuracy compared with the unimodal model approaches [5–11], but these cause discomfort and require experts' interpretation [12]. One alternative to the large number of sensors that are used for the complete PSG is the use of only EEG [4,13]. However, EEG recording requires the patients to wear scalp electrode caps, which are uncomfortable during sleeping. Another option is the combination of ECG, respiration, accelerometer (ACM) or actigraphy, which is an alternative to the EEG-based methods [14–19]. However, this increases the complexity of the recording and assessment. Few studies have investigated single-lead ECG-derived instantaneous heart rate-based sleep stage classification [9,20]. Compared with ECG, photoplethysmographic (PPG) is inexpensive, easy to wear and suitable for long-time monitoring. Clinically, PPG is a frequently used sensor to measure blood-oxygen saturation and heart rate. These physiological parameters are closely linked with sleep and hence have been proposed to identify two sleep stages: sleep and awake [21–26]. However, the accuracies reported in the above-mentioned studies suffer from biased sensitivity or specificity, which makes these unsuitable for computerized assessments. Research has also been reported where the binary assessment of sleep using PPG has been extended to multi-stage scoring [27]. These studies have classified the sleep period into three stages: awake, NREM and REM [28–31], and four stages: awake, light sleep (LS), deep sleep (DS) and REM [12,28,29,32,33]. For multi-stage sleep monitoring, previous methods have used either PPG and ACM or PPG, ACM and peripheral oxygen saturation (SpO2), while this study focuses on only PPG-based unimodal approaches. Additionally, for multi-stage sleep classification, deep learning-based models showed better performance [34,35] compared with the classical machine learning-based techniques; however, it suffered from limited explainability.

This research has investigated the use of a single-sensor—only PPG—recording for detecting the stages of sleep. Experiments were conducted where the four stages of sleep were identified by experts using PSG. The PPG recordings were then analysed to obtain surrogates for respiratory and cardiovascular parameters and a set of nonlinear features. The sleep labels provided by experts were used to train the machine-based classifiers for the classification of features obtained from PPG recordings.

The novelty of this work is that we have investigated the use of only PPG recordings to detect the four stages of sleep and have shown the effectiveness of using surrogates of arterial pressure for sleep stage detection obtained from PPG using hand-crafted features that have clinical interpretability of sleep physiology. This work has future applications for a single, wearable sensor-based sleep monitoring system.

## 2. Materials and methods

### 2.1. Database

The PSG data were recorded for the night sleep duration of 10 participants (nine male and one female, age 43–75 years). The length of the sleep time ranged from 6.8 to 10.1 h. All participants were volunteers and recruited from the outpatients at Charite Hospital, Berlin, Germany. All suffered sleep-disordered breathing and were free from a history of cardiac issues. The diagnosis was based on PSG outcomes and clinical symptoms. The research and data collection protocol were approved by the Charite Hospital Committee for Ethics in Human Research (2018), Berlin, Germany, and the experiments were conducted in accordance with the Helsinki declaration for ethical experiments, revised in 2013. A written consent was taken prior to the experiments. The demographic information of the subjects is shown in table 1. Each PSG recording included two-channel EEG (channel C3-A2 and C4-A1), ECG, PPG, left and right EOG, leg movements, thoracic and abdominal wall expansion, arterial oxygen saturation (SaO2) and oronasal airflow [36]. The details of the recorded signals are described in table 2. According to the American Academy of Sleep Medicine (AASM) criteria, the PSG recordings were segmented into 30 s non-overlapping epochs, and an expert sleep physiologist labelled sleep stages. The total number of sleep epochs was 9394. The details of the sleep stages of each subject are described in table 3.

**Table 1.** Demographic information of PPG–sleep dataset.

demographics	descriptions
number of subjects	10
sex (male/female)	9/1
range of age (years)	43–75
recording duration (hours)	7.7 + 1.23
total number of sleep epochs	9394

**Table 2.** Details of the recorded signals (sampling rate 128 samples s^−1^).

modality	no. of signal/equipment
EEG	two channels: C3-A2 and C4-A1
PPG	finger pulse oximeter
EOG	left and right EOG
EMG	single-channel EMG
ECG	single-channel ECG
leg movements	ACM
thoracic and abdominal wall expansion	respiratory inductive plethysmography belts
oronasal airflow	nasal pressure sensor
arterial oxygen saturation	finger pulse oximeter

**Table 3.** The details of the sleep stages of each subject. The total sleep segments vary from 815 to 1212. The total number of sleep epochs is 9394.

subject ID	total segments	wake	LS	DS	REM
01	1212	629	439	142	581
02	1212	795	214	87	301
03	924	245	331	243	574
04	909	388	288	134	422
05	909	202	434	221	655
06	872	192	482	108	590
07	864	102	448	177	625
08	844	108	608	107	715
09	833	500	168	105	273
10	815	247	356	121	477

### 2.2. Photoplethysmographic-based sleep stage classification

**Figure 1.**
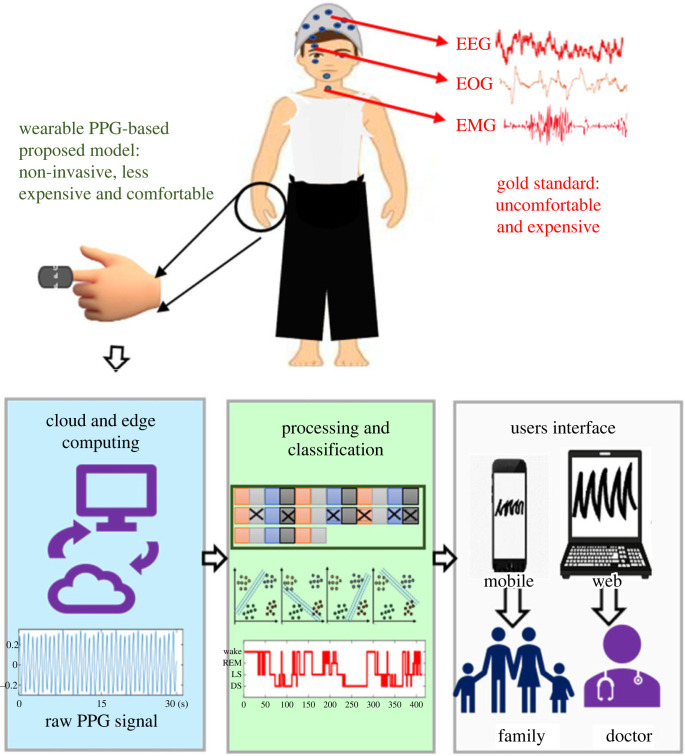
The block diagram of the PPG-based proposed model for multi-stage sleep classification with the PSG-based gold standard technique. The minimum number of required physiological signals for PSG are EEG, EOG and EMG which are demonstrated in red. Each subsection of the proposed model is shown in three different blocks with three different colours.

The PPG data were analysed and classified based on the sleep stages identified and labelled by the sleep experts using the PSG recordings. Pre-processing was performed to remove noise, and the denoised signal was analysed to extract 79 features that have been reported in the literature. The next step was the selection of the features, followed by classification. The block diagram of the proposed model is illustrated in figure 1. Each step of the model is described below:

#### 2.2.1. Pre-processing and segmentation

All PPG data were sliced into 30 s non-overlapping epochs as recommended by the American Academy of Sleep Medicine. After removing the baseline wander, each PPG epoch was smoothed with a Savitzky Golay FIR filter (order three, frame size nine samples). To reduce inter-experimental variability, PPG data were normalized based on *z*-score; zero mean and unit variance were used for feature extraction and classification. All epochs were scored as awake, LS, DS and REM based on the experts' labels of the sleep stages. For three-stage classification, LS and DS were merged and labelled as NREM. For binary sleep-wake classification, NREM and REM were merged and labelled as ‘sleep’.

#### 2.2.2. Feature extraction

It has been established that there are significant changes to the arterial blood pressure between the REM and NREM sleep phases [37]. Features of PPG that correspond to the changes in arterial blood pressure, such as the area under the PPG cycle, area of systolic and diastolic and the ratio of the systolic and diastolic area (complete list in table 4) were extracted from the recordings. The cardiac activity is regulated by the sympathetic and parasympathetic framework and induces heart rate oscillations at different rhythms during different sleep cycles [38]. Similarly, the respiratory pattern also changes during different sleep cycles [39]. To capture the changes of cardio-respiratory pattern during different sleep cycles, PPG peak-to-peak interval (PPI) features, which have been established to be surrogate of cardio-respiratory changes [38,40], were obtained from the recordings. The amplitude, intensity and frequency variations of PPG are associated with respiratory changes [40–42]. The PPG peaks were detected, and the PPI series were generated to compute these features. The power spectral analysis of the very low, low and high frequency of heart rate variability (HRV), which is an indirect measurement of sympathetic activity is included in the cardio-respiratory feature set.

**Table 4.** A list of features extracted from PPG after segmentation and pre-processing. *SD1_PPG_* represents SD1 feature extracted from PPG; *SD1_PPI_* represents SD1 feature extracted from PPI.

list of PPG features as surrogates for arterial blood pressure		
no. of feature	features	description of feature
1–7	mean *of A1, A2, A, T1, T2, IPAR, IPTR*	*A1*: systolic area, *A2*: diastolic area, *A*: area under PPG cycle, *T1*: systolic time, *T2*: diastolic time, *IPAR*: inflection point area ratio, *IPTR*: inflection point time ratio
8–14	s.d. of *A1, A2, Area, T1, T2, IPTR, IPAR*	
list of PPG features as surrogate of cardio-respiratory information		
15–19	mean of *PPI, RIAM, RIFM*	*PPI*: PPG peak-to-peak interval, *RIAM*: respiratory-induced amplitude modulation, *RIFM*: respiratory-induced frequency modulation
20–22	s.d. of *PPI, RIAM, RIFM*	
23–25	s.d. of successive difference of *PPI, RIAM, RIFM*	
26–28	root-mean-square of successive difference of *PPI, RIAM, RIFM*	
29–30	*pPP50,* s.e.m.	*pPP50*: per cent of PPI differences greater than 50 ms, s.e.m.: standard error of the mean PPI
*31–32*	*VLF, LF, HF, LF/HF*	*VLF*: very low-frequency power in the band 0.003–0.04 Hz, *LF*: low-frequency power in the band 0.04–0.15 Hz, *HF*: high-frequency power in the band 0.15–0.4 Hz, *LF/HF:* ratio of low-frequency and high-frequency power
list of statistical nonlinear-dynamical features extracted from both PPG and PPI series		
33–35	*avgAD_PPG_, stdAD_PPG_, MAD_PPG_*	mean absolute deviation, s.d. of absolute deviation, median absolute deviation of PPG signal
36–38	*IQR_PPG_, nCM_PPG_, avgE_PPG_*	interquartile range, *n*th central moment, average energy of PPG signal
39–41	*SF_PPG_, avgCL_PPG_, avgTE_PPG_*	shape factor, average curve length, average Teager energy of PPG signal
42–45	*GM_PPG_, HaM_PPG_, TM25_PPG_, TM50_PPG_*	geometric mean, harmonic mean, 25% trimmed mean, 50% trimmed mean of PPG signal
46–47	*Skew_PPG_, Kurt_PPG_*	skewness, kurtosis of PPG signal
48–51	*SD1_PPG_, SD2_PPG_, RSD1SD2_PPG_, CCM_PPG_*	Poincaré *SD1,* Poincaré *SD2,* ratio of *SD1* and *SD2,* CCM of PPG signal
52–54	*HA_PPG_, HM_PPG_, HC_PPG_*	Hjorth activity, Hjorth mobility, Hjorth complexity of PPG signal
55–57	*HDF_PPG_, LC_PPG_, KFD_PPG_*	Higuchi fractal dimension, Lyapunov coefficient, Katz fractal dimension of PPG signal
58–60	*avgAD_PPI_, stdAD_PPI_, MAD_PPI_*	mean absolute deviation, s.d. of absolute deviation, median absolute deviation of PPI series
61–63	*IQR_PPI_, nCM_PPI_, avgE_PPI_*	interquartile range, *n*th central moment, average energy of PPI series
64–66	*SF_PPI_, avgCL_PPI_, avgTE_PPI_*	shape factor, average curve length, average Teager energy of PPI series
67–70	*GM_PPI_, HaM_PPI_, TM25_PPI_, TM50_PPI_*	geometric mean, harmonic mean, 25% trimmed mean, 50% trimmed mean of PPI series
71–72	*Skew_PPI_, Kurt_PPI_*	skewness, kurtosis of PPI series
73–76	*SD1_PPI_, SD2_PPI_, RSD1SD2_PPI_, CCM_PPI_*	Poincaré *SD1,* Poincaré *SD2,* ratio of *SD1* and *SD2,* CCM of PPI series
77–79	*HA_PPI_, HM_PPI_, HC_PPI_*	Hjorth activity, Hjorth mobility, Hjorth complexity of PPI series

Pathological and age-associated changes of the nonlinear features such as fractal, entropy and Poincaré descriptors of cardiovascular signals have been established [43]. These indicate the change in the degrees of freedom of the system [43]. In this study, Higuchi fractal dimension (*HFD*), Katz's fractal dimension (*KFD*) and lower order to higher order statistical features were extracted from both PPG and PPI series. Average curve length (*avgCL*), shape factor *(SF)* and average Teager energy (*avgTE*) to measure the nonlinear dynamic of HRV and raw PPG were also obtained. These are listed in table 4.

Further to our earlier work, we employed the Poincaré descriptors (table 4) on the PPG and PPI series that are nonlinear and dynamical features obtained from the time series [44]. Poincaré plot is a geometrical representation of the time series into state-space by consecutively plotting the time series in the Cartesian coordinate. Four descriptors—s.d. of Poincaré plot perpendicular to the line-of-identity (*SD1*), along the line-of-identity (*SD2*), the ratio of *SD1* and *SD2*, and complex correlation measure (*CCM*) were calculated from Poincaré plot for lag *m* = 1. Poincaré *SD1* and *SD2* are associated with short-term and long-term variability, respectively. A total of 79 features were obtained from the recordings. To remove bias due to the difference in the features prior to classification, the recordings were detrended and normalized such that each feature had zero mean and unit variance.

#### 2.2.3. Feature selection

A set of distinct features improves the performance of data classification, but excessive numbers of redundant features can result in overtraining, increased error and computational complexity [45]. Statistical tests were conducted to identify the most suitable set of features from the whole feature set. The first step was to test the distribution of the data using Shapiro–Wilk test [46], and when the distribution was found not to be normal, the non-parametric distribution-free test was required to understand the significance of each feature. In this work, we have used the non-parametric Kruskal–Wallis test to identify significant features for two-stage sleep classification. The median value of the features for each class—those features having *p* < 0.05 were selected as these showed the statistically significant difference between the groups [47]. Kruskal–Wallis test results are unsuitable for identifying the features for separating the multi-class problem. To measure the capability of each feature in distinguishing the multi-classes, a multiple comparison test is used for computing the statistical significance in each pair of classes [48]. Thus, for both the three- and four-class classification, the statistical significance of the features was obtained from the following equation:

$$\mathrm{sigPair}\;=\overset{G_{T}}{\underset{i=0}{\sum }}s_{i}\;\mathrm{where}\;s_{i}=\{\begin{array}{ccc} 1 & \mathrm{if} & P(G_{x},G_{y})<0.05\\ 0 & \mathrm{otherwise}, & \end{array}\;\;\;$$

where *P*(*G_x,_ G_y_*) represents the *p*-value between any two sleep pairs. *G_T_* is the total number of sleep class pairs. There are three class pairs for three-stage classification: awake versus NREM, awake versus REM and NREM versus REM. There are six pairs for the four-stage classification: awake versus LS, awake versus DS, awake versus REM, LS versus DS, LS versus REM, and DS versus REM.

Features were selected during the training phase based on their statistically significant contribution to the group difference. The significance of the features was ranked based on *sigPair* value. These were considered as statistically significant for *p* < 0.05 for a minimum of two pairs for three-class classification and five pairs for the four-class classification. This resulted in the selection of 72, 64 and 50 statistically significant features for two-, three- and four-class classification, respectively. The set of ranked features that were selected based on the above for the four-stage classification are shown in table 5. Matlab 2021b (MathWorks) was used to perform all computations, including statistical analysis.

**Table 5.** List of selected features for four-stage sleep classification. Among 79 features, 50 features were statistically significant and used to train the classifiers. Among these, 12 are surrogate for arterial, 12 for cardio-respiratory and 26 are nonlinear-dynamical features.

sleep stage classification	feature type (no. of feature)	feature description and its *p*-value
four-stage sleep classification	morphological arterial blood pressure (12)	*mean of A1, A2, A, T1, T2, T, IPTR, IPAR* *s.d. of A, A2, T2, IPTR*
	surrogate cardio-respiratory (12)	*avgPPI, avgRIAM, avgRIFM, sdPPI, stdRIAM, stdRIFM,*
		*sdsdPPI, sdsdRIFM, sdsdRIAM, rmssdPPI, rmssdRIAM, rmssdRIFM*
wake versus LS versus DS versus REM	statistical nonlinear dynamical (26)	*avgAD_PPI_, MAD_PPI_, nCM_PPI_, TM25_PPI_, TM50_PPI_, avE_PPI_, SF_PPI_, svd_PPI_, KS_PPI_, HC_PPI_, avgAD_PPG_, MAD_PPG_, IQR_PPG_, nCM_PPG_, SF_PPG_, TM25_PPG_, avE_PPG_, SF_PPG_, SS_PPG_, KS_PPG_, SD1_PPG_, SD2_PPG_, RSD1SD2_PPG_, HC_PPG_, HFD_PPG_, sdtAD_PPG_*

### 2.3. Classification

Three machine-learning classifiers that have been reported in the literature for a similar problem [49] were used i.e. *K* nearest neighbour (KNN), support vector machine (SVM) and random forest. KNN is a non-parametric classification incorporating a regression algorithm. The classification is performed based on the most common class among the *K* nearest neighbours [50,51]. KNN requires the selection of distance measures and *K*-value. In this study, cubic and weighted Euclidean distances between the neighbours were considered, with *K* = 10 at first. In addition, the subspace KNN approach where the best *K*-value was derived from the data were applied. This corresponds to the lowest validation error from the training data. Finally, the data-driven *K*-value was used to determine the smallest number of features in the ensemble.

SVM is a broadly used supervised machine-learning technique that points out the optimal separating hyperplane to distinguish the data by maximizing the margin between the classes in the feature space [52]. The polynomial kernel was selected for the SVM because it was shown to have the highest classification [25].

Random forest is a supervised model that develops decision trees to classify the data. A bagged ensemble-based random forest classifier where each model is a decision tree was used in this study. This model incorporates all features for splitting a node instead of using random sampling—samples drawn with replacement, known as bootstrapping—which has been applied to build the trees in a classical random forest. The final classification was made by averaging the outcomes of each decision tree [53,54].

The focus of this work was not the choice of the classifier, but the use of the feature set for detecting the sleep stage and in that spirit, the use of KNN, SVM and random forest should be considered as one of the many options. Furthermore, with the relatively small size of the database, we did not investigate deep learning-based models.

### 2.4. Data management for training, model selection and cross-validation

The dataset was first randomly separated into the training set and the testing set: 70% for training and the remaining 30% for testing. This was repeated five times to remove the potential of bias and the results were averaged over the five repeats. *k*-fold cross-validation technique was used to identify the most suitable model, where the training set was divided into *k* = 10 subsets. Here, *k* − 1 subsets were used for training the model and the remaining set was used for validation. This process was repeated till each of the *k*-sets was used for validation, resulting in *k* models. The model with the best classification accuracy was selected and then tested with the dataset corresponding to the balance unseen 30% to evaluate its performance.

In addition to generic cross-validation, subject-specific performance measurement was recorded. Instead of using total sleep epochs from whole datasets, for subject-specific validation, training and testing sets were produced from the same subject where 70% samples of a participant were used for training and the remaining 30% for testing. This was repeated for each participant.

### 2.5. Performance metrics

As a performance metric, five statistical measures were chosen to evaluate the model performance: accuracy, sensitivity, specificity, precision and F-score. These were calculated as following:

$$\begin{array}{rl} & \mathrm{accuracy}\;=\frac{TP+TN\;}{TP+\;TN+FP+FN}\;100,\;\mathrm{sensitivity}=\frac{TP}{TP+\;FN}\;100,\\ & \mathrm{specificity}=\frac{TN}{TN+\;FP}\;100,\;F-\mathrm{score}\;=2\frac{\mathrm{sensitivity}\;\mathrm{specificity}\;}{\mathrm{sensitivity}+\mathrm{specificity}\;}\;100, \end{array}$$

where true positive (*TP*) is the number of sleep epochs detected as sleep, true negative (*TN*) is the number of awake epochs detected as awake, false positive (*FP*) is the number of awake epochs detected as sleep and false negative (*FN*) is the number of sleep epochs detected as awake.

To measure the strength of each feature, Spearman correlation coefficient was computed. The Spearman correlation coefficient of feature *X* and label *Y* is calculated as follows:

$$r\;(X,Y)=\frac{\sum_{i}^{n}(X_{i}-\bar{X})(Y_{i}-\bar{Y})}{\sqrt{\sum_{i}^{n}{\left(X_{i}-\bar{X}\right)}^{2}\sum_{i}^{n}{\left(Y_{i}-\bar{Y}\right)}^{2}}},$$

where $\bar{X}=\sum_{i}^{n}X_{i}/n$ and $\bar{Y}=\sum_{i}^{n}Y_{i}/n$, *n* is the length of each column. $\bar{X}$ and $\bar{Y}$ are the mean of *X* and *Y* respectively. The Spearman correlation coefficient *r* varies from –1 to +1. The value of *r* = + 1 indicates perfect positive correlation*, r* = −1 indicates perfect negative correlation, while *r* = 0 indicates no correlation between the feature and label.

## 3. Results

### 3.1. Performance evaluation

Feature selection showed that 72, 64 and 50 features were significant for two-, three- and four-class classification, respectively. For two classes (sleep and awake), the corresponding accuracy using subspace KNN, SVM and random forest was 83.75%, 84.66% and 85.22%, respectively. Three-class detection accuracy ranged between 78.30% to 82.62%, while for four stages of sleep, the overall accuracy was between 70.19% to 72.66%. Table 6 illustrates the performance metrics of the three classifiers for two, three and four stages of sleep classification.

**Table 6.** Performance evaluation of the proposed method for two class (wake, sleep), three class (wake, NREM, REM) and four class (wake, LS, DS, REM) classification using PPG signal.

two class (sleep-wake)							
methods	accuracy % (mean ± s.d.)	sensitivity (mean ± s.d.)	specificity (mean ± s.d.)	F-score (mean ± s.d.)	AUC (mean ± s.d.)		
subspace KNN	83.75 ± 0.85	87.79 ± 1.10	73.63 ± 2.45	80.01 ± 1.88	0.77 ± 0.011		
random forest	85.22 ± 0.62	87.86 ± 1.48	77.67 ± 3.26	82.45 ± 1.62	0.79 ± 0.010		
SVM cubic	84.66 ± 0.99	87.41 ± 1.27	77.79 ± 0.93	82.32 ± 0.90	0.79 ± 0.009		
three class (wake-NREM-REM)							
methods	avg Acc % (mean ± s.d.)	Acc wake (mean ± s.d.)	Acc NREM (mean ± s.d.)	Acc REM (mean ± s.d.)	F-score (mean ± s.d.)	AUC (mean ± s.d.)	
subspace KNN	79.31 ± 0.78	71.05 ± 1.64	85.41 ± 0.78	54.54 ± 3.60	65.90 ± 1.5	0.77 ± 0.005	
random forest	82.62 ± 1.21	77.67 ± 2.24	88.10 ± 1.17	36.36 ± 6.07	62.28 ± 1.93	0.79 ± 0.002	
SVM cubic	79.62±	74.45 ± 1.44	82.52 ± 1.70	62.80 ± 4.10	65.70 ± 2.15	0.78 ± 0.005	
four class (wake-LS-DS-REM)							
methods	avg Acc % (mean ± s.d.)	Acc wake (mean ± s.d.)	Acc LS (mean ± s.d.)	Acc DS (mean ± s.d.)	Acc REM (mean ± s.d.)	F-score (mean ± s.d.)	AUC (mean ± s. d.)
subspace KNN	70.19 ± 0.32	74.85 ± 1.10	69.02 ± 0.96	67.47 ± 1.73	48.76 ± 2.63	60.05 ± 2.27	0.66 ± 0.001
random forest	72.39 ± 0.48	79.00 ± 0.79	73.98 ± 1.77	58.82 ± 1.14	34.71 ± 3.83	60.78 ± 2.64	0.68 ± 0.002
SVM cubic	72.23 ± 0.38	74.70 ± 0.88	71.50 ± 1.90	62.63 ± 1.54	61.90 ± 3.23	62.22 ± 3.44	0.68 ± 0.001

Acc: accuracy, s.d.: standard deviation, AUC: area under the curve.

The performance without the surrogate of the arterial blood pressure features is shown in table 7. A comparison of this with table 6 shows that the inclusion of arterial blood pressure features improved the model classification accuracy by about 3% for each class as well as overall classification, but with variations, and showed significant improvement in REM stage classification. It is observed from table 6 that for both three- and four-stage sleep classification, the accuracy of REM classification was the lowest. This may be attributed to the training bias because of the small number of REM samples compared with other classes. It is also seen that the random forest classifier shows the single highest overall accuracy. SVM with a polynomial (cubic) kernel shows consistent results over all the KNN options and random forest for each sleep stage classification. This may be ascribed to SVM being more resilient to the class imbalanced dataset. In addition to polynomial (cubic) kernel, we also applied linear and Gaussian kernel. We only reported the results for SVM with polynomial kernel since it outperformed the other kernels.

**Table 7.** Performance evaluation of the proposed method for two class (sleep, wake), three class (wake, NREM, REM) and four class (wake, LS, DS, REM) classification without surrogate arterial blood pressure features.

two class (sleep-wake)							
methods	accuracy % (mean ± s.d.)	sensitivity (mean ± s.d.)	specificity (mean ± s.d.)	F-score (mean ± s.d.)	AUC (mean ± s.d.)		
subspace KNN	81.18 ± 0.52	91.52 ± 0.82	67.72 ± 1.65	77.84 ± 1.28	0.77 ± 0.006		
random forest	81.68 ± 1.09	89.80 ± 1.18	70.70 ± 3.13	79.11 ± 1.75	0.79 ± 0.014		
SVM cubic	81.20 ± 0.58	88.86 ± 1.81	72.76 ± 2.18	80.01 ± 0.73	0.79 ± 0.005		
three class (wake-NREM-REM)							
methods	avg Acc % (mean ± s.d.)	Acc wake (mean ± s.d.)	Acc NREM (mean ± s.d.)	Acc REM (mean ± s.d.)	F-score (mean ± s.d.)	AUC (mean ± s.d.)	
subspace KNN	77.33 ± 1.10	70.09 ± 1.80	86.31 ± 1.10	46.28 ± 3.80	62.18 ± 1.5	0.73 ± 0.006	
random forest	78.51 ± 1.40	74.65 ± 3.30	87.72 ± 1.66	36.36 ± 5.50	61.80 ± 1.93	0.74 ± 0.004	
SVM cubic	77.59 ± 1.02	73.06 ± 2.20	83.56 ± 1.90	60.33 ± 4.40	63.98 ± 1.58	0.73 ± 0.005	
four class (wake-LS-DS-REM)							
methods	avg Acc % (mean ± s.d.)	Acc Wake (mean ± s.d.)	Acc LS (mean ± s.d.)	Acc DS (mean ± s.d.)	Acc REM (mean ± s.d.)	F-score (mean ± s.d.)	AUC (mean ± s.d.)
subspace KNN	68.11 ± 1.65	74.35 ± 1.40	70.02 ± 1.68	57.47 ± 2.35	51.23 ± 4.33	59.56 ± 3.70	0.61 ± 0.002
random forest	69.65 ± 1.35	74.05 ± 1.20	72.83 ± 1.60	58.82 ± 2.77	38.83 ± 3.33	59.63 ± 2.20	0.62 ± 0.003
SVM cubic	69.38 ± 1.54	76.02 ± 1.45	68.14 ± 1.96	59.51 ± 1.93	51.23 ± 2.54	60.62 ± 2.44	0.62 ± 0.002

Acc: accuracy, s.d.: standard deviation, AUC: area under the curve.

**Figure 2.**
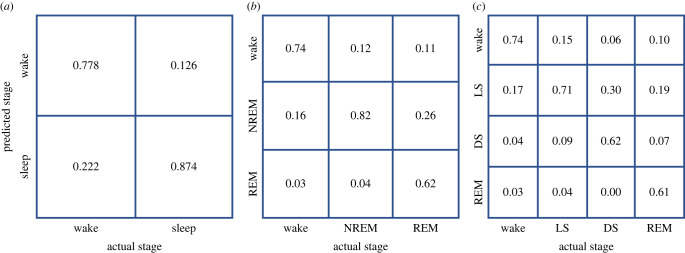
Confusion matrix of support vector machine classifier for two- (*a*), three- (*b*) and four-stage (*c*) sleep classification.

The confusion matrices of the SVM classifier for the two, three and four classes are shown in figure 2. It is observed that the overall accuracy for the two-stage sleep classification is higher than that of three- and four-sleep stage classification. This can be attributed to the lack of difference in the cardiac and respiratory signals between the awake versus LS and REM versus LS stages. In the case of four-stage classification, 17.7% awake classes were detected as LS and 15.4% LS were detected as awake. Similarly, 19.8% and 10.7% of REM were falsely classified as LS and awake, respectively. This shows that the accuracy of multi-stage sleep classification is lower than that of two-stage classification.

### 3.2. Sleep scoring for subject-specific multi-night sleep

**Figure 3.**
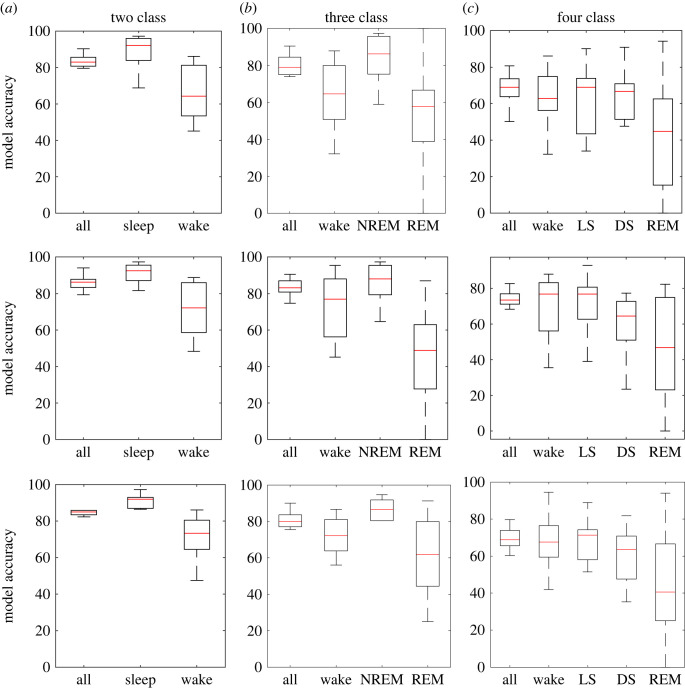
Performance of a subspace KNN (top row), random forest (middle row) and SVM (bottom row) classifier for different staging resolutions. Interquartile range plots of model performance obtained from subject-specific cross-validation for (*a*) two-class sleep versus awake classification, (*b*) three-class awake versus NREM versus REM classification and (*c*) four-class awake versus LS versus DS versus REM classification.

**Figure 4.**
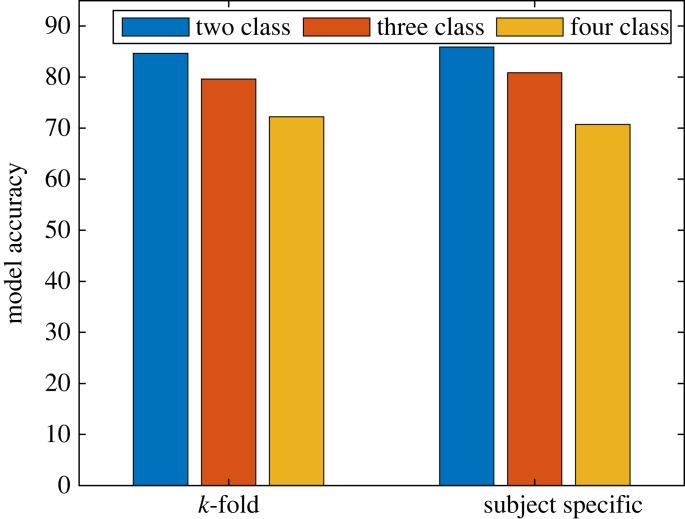
Model accuracy of two-, three- and four-stage sleep classification for generic and subject-specific cross-validation using SVM classifier with the polynomial kernel.

The overall classification accuracy of two-, three- and four-stage sleep classification using subspace KNN was 84.36%, 80.12% and 68.01%, using random forest was 86.39%, 83.15% and 73.05%, and using SVM was 85.86%, 80.87% and 70.75, respectively, for subject-specific cross-validation. The model performance of each classifier for each stage is demonstrated in figure 3. Like generic *k*-fold cross-validation, random forest shows the single highest overall accuracy than KNN and SVM for subject-specific cross-validation. In terms of each stage classification, SVM with polynomial (cubic) kernel shows consistent results over KNN and random forest that is reflected by the lower interquartile range of model accuracy of each stage (figure 3). The model accuracy of different sleep stages using the SVM classifier for 10-fold and subject-specific cross-validation are shown in figure 4.

## 4. Discussion

This work has investigated the use of PPG for multi-stage sleep classification using a machine-learning approach. PPG is a single wearable sensor that an individual can use without requiring a clinical expert to mount the sensors. The current methods use several sensors to record physiological parameters such as cardiac activities and respiration during sleep. By contrast, the proposed method will replace these by obtaining surrogate features from the PPG recordings (table 4). The sympathetic and parasympathetic systems [55] manage heart rate rhythms and respiratory activity during the different sleep stages [39]. Surrogate cardiac and respiratory parameters obtained from the PPG recordings were the basis of this study. These were augmented with nonlinear features extracted from the recordings using Poincaré analysis.

**Figure 5.**
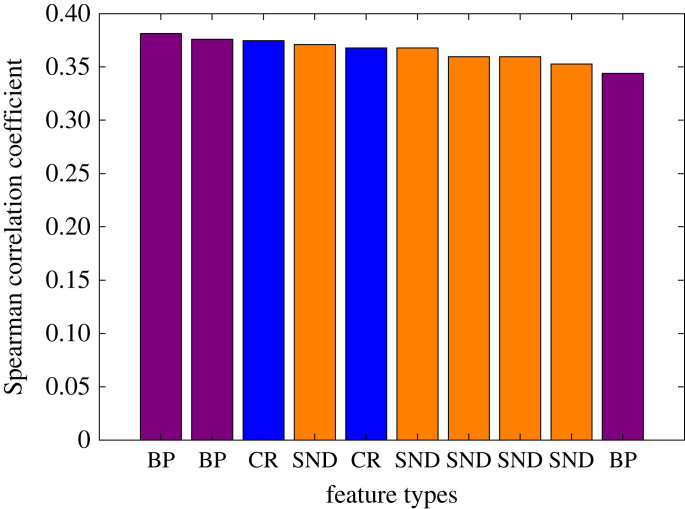
The rank of the top 10 features using Spearman correlation coefficient. BP, CR and SND are the features from surrogate arterial blood pressure, cardio-respiratory and statistical nonlinear-dynamical feature, respectively.

There is a strong relationship between blood flow and metabolism in the central nervous system with sleep; sleep modulates these using central autonomic control and regional blood flow at all levels of the nervous system [56]. Blood pressure decreases progressively during relaxed wakefulness, which is reversed during LS, but with further reduction during DS. By contrast, blood pressure increases in the form of phasic hypertensive events in REM sleep [57]. This indicates that blood pressure has a close association with the sleep stages and supports the observation of this research that the incorporation of surrogate arterial blood pressure improves the classification of different sleep stages. This study has investigated 16 PPG features that are associated with arterial blood pressure [58] for sleep-stage classification. The results show that the inclusion of these features improved the classification accuracy from 81.20% to 84.66%, 77.59% to 79.62% and 69.38% to 72.23% for two-, three- and four-class classification, respectively (tables 6 and 7). This is also confirmed by the membership of the highest ranked features shown in figure 5.

The results show that there were 50 statistically significant features for four-stage sleep classification, and 12 of these were surrogates for arterial blood pressure. Spearman correlation coefficient of the significant features shows that among the top 10 best features, three features are surrogates for arterial blood pressure, two for cardio-respiratory, and there are five statistical nonlinear-dynamical features, with arterial blood pressure markers being the top two (figure 5).

To establish the efficacy of using PPG for sleep-stage classification, a comparison was performed with the gold standard for sleep monitoring. The results show that EEG and ECG outperformed PPG for multi-stage sleep assessment [59,60]. While PPG can provide surrogate measures, EEG is the direct measurement. However, it requires precise electrode placement and the participant to wear the electrode cap, making them impractical for home-based sleep monitoring.

A subject-specific model was developed to reduce the effect of intra-participant variability and these results are shown in figure 3 and 4. These indicate that PPG could be a good alternative of PSG-based multi-night sleep monitoring of an individual. It is envisaged that on the first night could be PSG-based monitoring, using which the PPG model could be developed, and this could be used for PPG-only-based subsequent night sleep monitoring.

A comparison was performed with the other studies that have proposed the use of PPG for sleep monitoring and this is shown in tables 8 and 9. From table 8, it is seen that our proposed model outperforms all other models for two-stage sleep classification enlisted except [28,30], which, however, also included tri-axial ACM signal along with PPG. Moreover, the sensitivity or specificity of [28,30,60] was lower than our results. Our model had the balanced overall performance based on sensitivity, specificity and accuracy among all PPG-based work reported in the literature.

**Table 8.** The comparison of the proposed model with the existing studies in literature for two class (sleep-wake) classification.

methods	two class (sleep-wake)			
	accuracy (%)	sensitivity (%)	specificity (%)	cross-validation
Dekhordi *et al*. [21]^b^	77	78	72	own dataset
Motin *et al.* [23]^b^	72.36	70.64	74.22	70/30
Dekhordi *et al.* [24]^b^	77	80	70	independent dataset
Fonseca *et al.* [28]^a^	91.50	58.20	96.90	independent dataset
Walch *et al.* [30]^a^	90	93	60	Monte Carlo cross-validation and leave-one-out
Ucar *et al.* [49]^b^	73.36	81	77	*k*-fold
Habib *et al*. [34]	94.4	94.4	94.0	leave-one-subject-out
Eyal & Baharav [60]^b^	84.30	38.10	91.70	independent dataset
proposed^b^	84.66	87.41	77.79	random 70/30 split with five repetition

^a^Both PPG and ACM signals are used for sleep-wake classification.

^b^Only PPG signal is used for sleep-wake classification.

**Table 9.** The performance comparison (in terms of accuracy as performance metric) of the proposed model with the existing studies in literature for three-class (wake-NREM-REM) and four-class (wake-LS-DS-REM) classification.

method	three class (wake-NREM-REM)				four class (wake-LS-DS-REM)					cross-validation
	overall (%)	wake (%)	NREM (%)	REM (%)	overall (%)	wake (%)	LS (%)	DS (%)	REM (%)	
Fonseca *et al*. [28]^b^	72.90	—	—	—	59.30	—	—	—	—	independent dataset
Molkkari *et al*. [29]^d^	72.50	—	—	—	60.10	—	—	—	—	leave-one-subject-out
Walch *et al*. [30]^b^	72.30	—	—	—	—	—	—	—	—	Monte Carlo cross-validation and leave-one-out cross-validation.
Zhang *et al*. [31]^a^	75.10	—	—	—	—	—	—	—	—	48 patients
Beattie *et al*. [32]^b^	—	—	—	—	69	69.3	69.2	62.4	71.6	leave-one-subject-out
Fedorin *et al*. [33]^b^	85	—	—	—	77	—	—	—	—	leave-one-subject-out
Wu *et al*. [12]^c^	78				62					leave-one-subject-out
Habib *et al*. [34] ^d^	94.2	95.0	94.0	90.0	92.9	95.0	91.0	93.0	92.0	leave-one-subject-out
Korkalainen *et al*. [35]^d^	80.10				68.50					80:10:10 (89 recordings)
proposed^d^	79.62	74.45	82.52	62.80	72.23	74.70	71.50	62.63	61.90	random 70/30 split with five repetition

^a^PPG, ACM and SpO2 signals are used for the classification.

^b^PPG and ACM signals.

^c^PPG, and SpO2 are used for the classification.

^d^Only PPG signal is used for sleep stage classification.

From table 9, it is observed that most previously reported PPG-based multi-stage sleep classification also used ACM recordings [28,30–33] or SpO_2_ [12,28] while the model tested in this paper only used PPG. The proposed single-sensor PPG-based multi-stages classification performance was similar or comparable to the other works, even though these had multiple sensors [28–33]. Our model outperformed the existing models [28–33] in terms of the accuracy of each stage except [33–35] where [34,35] are deep learning-based models that have limited explainability.

One limitation of this study is that it is based on a single night sleep data of 10 participants who had visited the sleep clinic with sleep problems. This is comparable with many other studies; however, the number of participants is relatively small, and this does not allow for investigating differences due to demographics or other health conditions. There is also the potential of bias because all the participants had reported sleep disorders. The other limitation of this study is that being a single night data, and it cannot be tested for repeatability.

## 5. Conclusion

This work has demonstrated the feasibility of an automated multi-stage sleep classification using only PPG with an overall accuracy of 84.66%, 79.62% and 72.23% for two-, three- and four-stage sleep classification, respectively, for patients with sleep disorders. Since PPG can be acquired unobtrusively and non-invasively, it is suitable for long-term routine sleep monitoring without troubling the normal sleep of the individuals. This work has demonstrated the potential of a computerized, pulse oximetry-based sleep screening with a more detailed epoch-by-epoch investigation, comparable to the one given by PSG. However, further validation of the model on a large clinical population with broad pathological profiles repeated over several nights is necessary to obtain clinical evidence and advance this work for translation. This work has advanced the use of PPG-based sleep-stage classification as an alternative for the current PSG-based sleep-stage assessment.

## Data Availability

Data available from the Dryad Digital Repository: https://doi.org/doi:10.5061/dryad.1rn8pk0z4 [61]. https://doi.org/10.6084/m9.figshare.21981998 [62]. https://doi.org/10.6084/m9.figshare.21981983 [63].
